# Reduced translucency and the addition of black patterns increase the catch of the greenhouse whitefly, *Trialeurodes vaporariorum*, on yellow sticky traps

**DOI:** 10.1371/journal.pone.0193064

**Published:** 2018-02-15

**Authors:** Clare Sampson, Anca D. Covaci, James G. C. Hamilton, Nayem Hassan, Shakir Al-Zaidi, William D. J. Kirk

**Affiliations:** 1 Centre for Applied Entomology and Parasitology, School of Life Sciences, Huxley Building, Keele University, Newcastle-under-Lyme, Staffordshire, United Kingdom; 2 Russell IPM Ltd, Deeside Industrial Park, Flintshire, United Kingdom; 3 Division of Biomedical and Life Sciences, Lancaster University, Lancaster, United Kingdom; Chinese Academy of Agricultural Sciences, CHINA

## Abstract

The greenhouse whitefly *Trialeurodes vaporariorum* (Westwood) (Hemiptera: Aleyrodidae) is a pest of a wide range of vegetable and ornamental crops in greenhouses around the world. Yellow sticky traps are highly attractive to flying adults and so are frequently used to monitor the pest. Our aim was to test whether changes in trap translucency or the addition of printed black patterns could increase the catch on yellow sticky traps in greenhouses. Field trials were carried out in commercial crops of strawberry and tomato under glass over three years. Reduced trap translucency increased trap catches by a factor of 1.5 to 7.0 and the catch increased significantly for both females and males. Spectrometer measurements showed that the increased catch was consistent with an increased landing stimulus from a colour opponency mechanism i.e. the ratio of energy from 500–640 nm to the energy from 300–500 nm. Printed black patterns increased trap catches on specific types of trap, by a factor of 1.4 to 2.3, and the catch increased significantly for both females and males. The patterns increased trap catch on moderately translucent traps, but decreased trap catch on less translucent traps. The evidence points to a contrast/edge effect of pattern, but laboratory experiments are needed to clarify this. Exploitation of these translucency and pattern effects could improve the efficacy of yellow traps for monitoring and mass trapping in crops.

## Introduction

It has long been known that yellow sticky traps (yellow card or plastic covered with a thin layer of a clear glue) or yellow water traps (yellow dishes filled with water and a little detergent) are highly attractive to many insects and so can be used for monitoring crop pests [1–3], mass trapping [2,4] or biodiversity surveys [5,6]. One of the earliest demonstrations of the effectiveness of yellow traps was by Lloyd in 1921 [7], who found that adults of both sexes of the greenhouse whitefly, *Trialeurodes vaporariorum* (Westwood) (Hemiptera, Aleyrodidae) were particularly attracted to yellow cards. Yellow sticky traps are now widely used commercially as part of Integrated Pest Management (IPM) in greenhouses, mainly for monitoring pest aphids, whiteflies, thrips and small flies [1,2]. Millions of sticky traps are used worldwide, so improved yellow traps would be of considerable benefit for pest management.

Yellow traps are particularly effective for leaf-feeding insects and flower-visiting insects, although the latter are often attracted to other colours as well [8–10]. The best explanation of the response to yellow is that in many leaf-feeding insects the landing stimulus is determined by a ‘colour opponency mechanism’ with positive input from a green receptor and negative input from a blue and perhaps also a UV receptor [11–13]. As a result, the strongest landing stimulus is from surfaces that have high reflectance in the positive long-wave region from green to red (about 500–640 nm) and low reflectance in the negative short-wave region from UV to blue (about 300–500 nm). Yellow-reflecting surfaces show the highest ratio of long-wave to short-wave and so catch more than green, blue or white surfaces. This suggests that a yellow trap is a ‘supernormal foliage stimulus’ [8,11]. Experiments with light-emitting diodes (LEDs) or monochromatic light have shown that many leaf-feeding insects have a peak response to green light (520–560 nm), which is consistent with the colour opponency theory [11,12,14]. The peak response wavelength (green) does not predict the colour of the most attractive coloured surface (yellow) because the landing response is affected by a wide range of wavelengths, not just the peak wavelength.

Studies of the effectiveness of sticky traps have mainly focused on wavelength [15,16], but other features of the trap, such as background [17–20], length of edge [21,22], shape [18–20], position [23–25], and surface texture [26] can also be important. The development of LEDs allows the selection of particular wavelengths and is opening up new possibilities for trapping in conjunction with sticky traps, with the advantage that they are not dependent on ambient light conditions [14,27]. However, sticky yellow traps without LEDs provide a simple, low-tech approach that is therefore likely to remain in widespread use.

In this paper, we investigated ways of improving the effectiveness of yellow sticky traps for *T*. *vaporariorum* in greenhouses. This species is a pest of a wide range of vegetable and ornamental crops in greenhouses, including tomato, cucumber, strawberry and poinsettia, in Europe and around the world, and damage is caused by feeding, contamination with honeydew and sooty mould, and the transmission of viruses [28,29]. We did not include the silverleaf whitefly *Bemisia tabaci* (Gennadius) in our study because it does not occur in the United Kingdom where the experiments were carried out.

*T*. *vaporariorum* is particularly caught on yellow sticky traps [7,16,26,30,31]. It is a dichromat with sensitivity in the yellow-green (peak 520 nm) and the UV (peak 340 nm) and this, together with its responses to wavelengths in the laboratory, provides supporting evidence for the operation of the colour opponency mechanism [32–34]. Responses change according to the flight status of the individual: adults at the start of a long-duration flight fly towards UV; adults at the end of a long-duration (migratory) flight show a ‘fall reflex’ over yellow-reflecting surfaces; and adults on subsequent short-duration (non-migratory) flights orientate towards vertical yellow surfaces and away from UV [12,32,35,36].

In our field trials with commercial yellow sticky traps in the form of roller traps (rolls of plastic sheet, typically 30 cm high that unroll to a length of 100 m) and small plastic boards (typically 10 cm x 25 cm), we noticed that double thickness traps, which had low translucency, caught more whiteflies than single thickness traps, which were more translucent. This was observed with both *B*. *tabaci* in Spain and *T*. *vaporariorum* in England. Initial trials also suggested that black patterns printed on yellow sticky traps could increase total trap catch. We hypothesised that this could be because the patterns have long contrasting edges, which have a stimulatory effect on insect compound eyes [37]. This effect could be why some insects land particularly at trap edges [22,38]. Alternatively, it could result from an optomotor response to the black lines. For example, Kennedy et al. [13] described how aphids ‘hesitated and hovered’ a few centimetres in front of vertical black stripes. Such an effect could perhaps increase trap catch in and around a pattern of black stripes. A third possibility is that a black pattern makes the trap darker on average and so is having the same visual effect as making the trap less translucent.

The aim of our study was to investigate these observations and test whether reduced translucency (increased opacity) and printed black patterns could increase the catch of *T*. *vaporariorum* on yellow sticky traps in greenhouses, thus gaining further insight into visual responses to traps in the field and suggesting ways of improving the efficacy of yellow traps for monitoring and mass trapping in crops.

## Materials and methods

### Field trials

Field experiments were carried out on private land and we confirm that the owner of the land gave permission to conduct the study on the site.

### Effects of trap translucency

Experiment A (thickness of trap type R1). To determine the effect of trap translucency on whitefly catch, we compared adjacent single and double thicknesses of a yellow roller trap (30 cm height) with moderate translucency (trap type R1). Such traps are widely available commercially. Doubling the thickness lowered the translucency and this was quantified with a spectrometer (see below). The experiment was carried out in a commercial strawberry crop (*Fragaria* x *ananassa ‘*Lusa’) in a glasshouse in central England (N 53° 41.74’ W 2° 52.66’). The crop was grown on raised gutters with drip irrigation and was flowering and fruiting. A 4 m length of trap was suspended between two greenhouse posts along a crop row, with the trap surfaces vertical and the bottom of the trap 10 cm above the top of the canopy. The central part of the trap was divided into six paired trap sections (15 cm wide) with each pair having a single thickness section and a double thickness section. The order of treatments within the pair was randomly allocated to give a randomised complete block design. Double thickness was created by cutting out sections of trap (15 cm x 30 cm) from an unused part of the same trap and sticking one on each section allocated the double thickness treatment. The trap was left out for 7 days in April 2016. Whiteflies were counted on both sides of the central 10 cm x 30 cm part of each trap section. The experiment was repeated in the same crop at the same stage over 22 days in March/April 2017 to obtain further data and also test the effect of trap translucency on each sex and on the distribution of whiteflies on the trap. The replication was increased by using a 12 m length of trap with nine paired trap sections (30 cm wide). Whiteflies were counted separately for each sex on both sides of the whole of each section (30 cm x 30 cm).

Experiment B (trap types R1 and R2). As a further test of whether the increase in trap catch was due to the reduction in trap translucency, the original trap (trap type R1) was compared with a less translucent (more opaque) trap of the same height (30 cm) and thickness, manufactured with an increased amount of the same yellow dye to reduce translucency (trap type R2). The experiment was a randomised complete block design with nine blocks and one replicate per block of each of the two types of trap, in the same crop as in experiment A. Each trap was a 3 m length of roller trap secured between greenhouse posts along a crop row, with the trap surfaces vertical and the bottom of the trap 10 cm above the top of the canopy. Traps were 4 m apart within blocks and blocks were a minimum of 4 m apart, with all traps in the same orientation. The traps were left out for 7 days in April 2016. Whiteflies were counted on the whole of each trap between the greenhouse posts.

Experiment C (thickness of trap type R2). The above experiment with single and double thicknesses of yellow trap (experiment A) was repeated in the same way for the less translucent trap (trap type R2), except that there were eight paired trap sections (15 cm wide). The traps were left out in the same crop for 9 days in April/May 2016.

### Trap patterns

**Fig 1:**
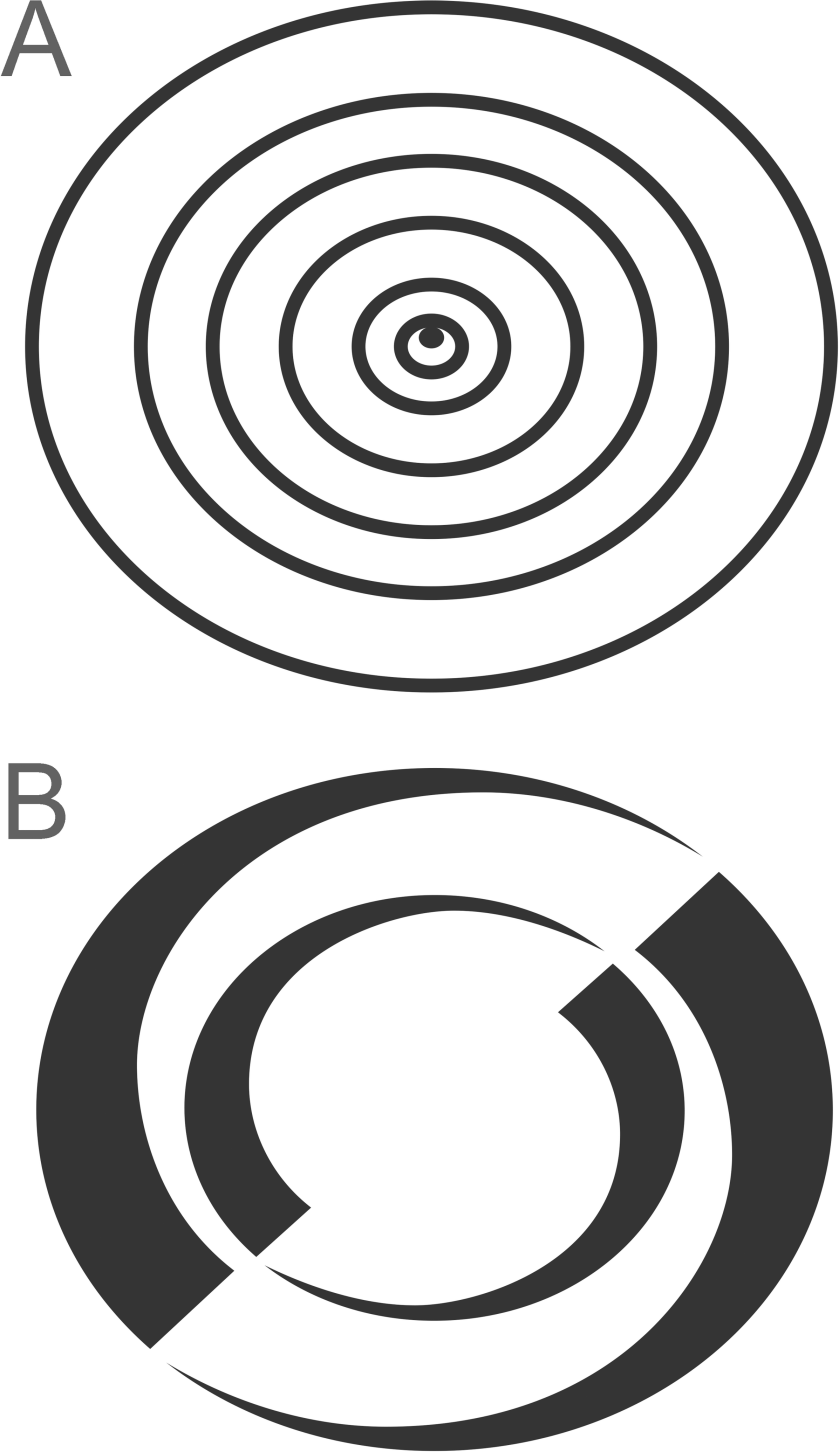
Patterns printed on traps. (A) Pattern A (uniform ovals), width 80 mm, height 69 mm. (B) Pattern B (tapered ovals), width 80 mm, height 69 mm.

Two patterns were used in our experiments. These were printed on the traps using black ink (Pantone black C). The patterns were printed centrally on one side only of the trap to allow us to test whether the effect on trap catch was on the pattern side or the plain side or both. We did not investigate the features of the pattern that affected the response, so we chose two patterns that would present contrasting edges at several distances apart to whiteflies from whatever angle they approached. Pattern A (uniform ovals) consisted of six concentric ovals of line width 2 mm with spacings of 10 mm, 7 mm and 4 mm, and an off-centre dot near the middle. The entire pattern was 80 mm wide and 69 mm high (Fig 1A). Pattern B (tapered ovals) was the same size and consisted of two concentric ovals increasing gradually in width from 0 mm to 10 mm with a spacing of 12 mm (Fig 1B). A single pattern A was printed on patterned board traps, whereas a row of pattern B at a repeat interval of 180 mm was printed down the middle of patterned roller traps.

### Effects of trap patterns

The following experiments (D-F) were carried out in a commercial tomato crop (*Solanum lycopersicum*) in a glasshouse in central England (N 53° 41.70’ W 2° 54.82’). The crops were grown hydroponically in rockwool blocks and supported on strings, and were flowering or fruiting or both. The experiments were randomised complete block designs with one replicate of each treatment per block. Each trap was suspended vertically with the bottom of the trap (the 10 cm side) about 10 cm above crop height by attaching it with a wooden clothes peg to a vertical string supporting the crop. In each experiment, traps were orientated so that the pattern side and the corresponding plain side on the control faced the same direction. The choice of trap spacing was influenced by plant spacing and layout and the desirability of keeping traps in areas of similar whitefly density, which was assessed by inspection.

Experiment D (pattern and trap size). To test whether patterns affected trap catch and whether the relative area of the pattern on the trap made a difference, we compared two sizes of yellow sticky board traps (large: 10 cm x 25 cm; small: 10 cm x 12.5 cm), with and without a centrally positioned pattern (pattern A). Large traps were 5% black and small traps were 10% black by area on the pattern side. There were 20 replicate blocks. All the traps were placed along one crop row, aligned parallel to the row, facing west, with 60 cm between traps within blocks and a minimum of 1 m between consecutive blocks. The traps were left out for 2 days in October 2015. Whiteflies were counted separately on each side of the traps.

Experiment E (pattern and distribution of catch). To test where on the trap the pattern increased trap catch, we compared the number of whiteflies on the top, middle and bottom of plain and patterned (pattern A) yellow sticky board traps (10 cm x 25 cm). There were 17 replicate blocks. Traps were aligned parallel to the crop rows, with 2 m between traps within blocks and a minimum of 3.5 m between blocks. The traps were left out for 24 h in October 2014. Whiteflies were counted separately on the top, middle and bottom thirds of the glue-covered area on each side of the traps (each third was 7 cm x 10 cm). The pattern was printed entirely within the middle section of the east-facing front side of the pattern traps.

Experiment F (pattern and shade of grey). To determine whether the shade of grey and degree of contrast affected response to pattern, we compared the effects of pattern A in three different shades of grey or no pattern, on yellow sticky board traps (10 cm x 25 cm). The shades were light grey (Pantone 400C), mid grey (Pantone 403C) and black (Pantone black C). There were 20 replicate blocks. Traps were aligned parallel to the crop rows, facing east, with 60 cm between traps within blocks and a minimum of 1 m between consecutive blocks along a row and a minimum of 2 m between blocks on different rows. The traps were left out for 7 days in September 2015. Since only the black pattern and not the mid grey or light grey patterns showed through to the other (non-pattern) side, whiteflies were counted only on the pattern side and the corresponding side facing the same direction on the traps with no pattern. To test whether catches of both sexes of whitefly were increased by a pattern, the sexes were counted separately on the control traps with no pattern and the traps with the black pattern.

### Effects of trap translucency on response to trap pattern

Experiment G (translucency and pattern). To determine whether trap translucency affected the whitefly response to pattern, the trap catch was compared on traps with and without pattern B (Fig 1B) on sections of yellow roller trap (30 cm height) with moderate translucency (trap type R1) and on sections of less translucent yellow roller trap (trap type R2). The experiment was carried out in the same glasshouse strawberry crop as for experiments A-C. The four treatments were laid out in a randomised complete block design with 10 blocks and one replicate per block. Each trap was above a different crop row, aligned perpendicular to the crop row, with 1 m (row spacing) between traps within a block and a minimum of 1 m between consecutive blocks. Sections of the roller traps (width 46 cm, height 30 cm) were suspended with the surfaces vertical and the bottom of the trap about 10 cm above crop height by attaching them with staples to horizontal crop wires above the crop. All traps were orientated so that the pattern side and corresponding plain side on the control faced in the same direction (SSW). The traps were left out for 24 h in July 2016.

### Counting whiteflies on traps

At the end of experiments, all traps were wrapped in thin, clear plastic (cling film) and stored in a -20°C freezer. The number of whiteflies per trap was counted in the laboratory using a x7 magnification head lens. The sexes were distinguished on the traps by examination of the end of the abdomen under a dissecting microscope. Males have distinctive tong-like parameres, whereas the females either have an extended ovipositor or have a characteristic blunt shape caused by the retracted ovipositor [39]. If the wings obscured the abdomen, either the wings were moved apart or the wax was rubbed off gently so that the abdomen tip could be seen through the wings.

### Spectrometer measurements

Percentage diffuse reflectance spectra were measured from 300–700 nm with a FLAME-S-UV-VIS spectrometer with an ISP-50-8-R-GT integrating sphere attachment and a UV-VIS-NIR light source (all from Ocean Optics, Duiven, the Netherlands). We used a WS-1-SL Spectralon reflectance standard (Ocean Optics, Duiven, the Netherlands). Multiple trap thicknesses were measured to show the effect of the trap material only and prevent any effect of the background. Translucency was measured as transmittance, which is defined as the proportion of the light energy falling on a body that is transmitted through it. Percentage transmittance spectra were measured with the above spectrometer and light source. The probe and light source were positioned as close as possible to each other on either side of the trap. Absolute irradiance spectra were measured with a FLAME-S-UV-VIS spectrometer calibrated for absolute irradiance (Ocean Optics, Duiven, the Netherlands), with sunlight from a clear sky and the sensor positioned perpendicular to the vertical trap surface at a distance of 50 mm. The side of the trap facing the sensor was defined as the front of the trap and the side away from the sensor was defined as the back. Measurements were taken with the sun shining on the front and with the sun shining on the back.

### Data analysis

Data for analysis of variance were transformed to log_10_(*x*), or log_10_(*x*+1) if any zeroes were present, to homogenise the variance. Residuals were checked for normality. Multiple comparisons were made using Tukey’s test. A general linear model was used for regression analysis, allowing for blocks. The data for the regression were transformed to log_10_(*x*+1) and the residuals were checked for normality. Regression on increasing shades of grey used a dummy variable where plain = 0, light grey = 1, mid grey = 2, and black = 3. All tables and figures show untransformed means and standard errors for adult whiteflies per trap, whereas all the statistical analyses used log-transformed data and allowed for blocks. Statistical analysis was carried out with Minitab 16 (Minitab Inc., USA) and measurement of the areas under curves was carried out with Origin 2016 (OriginLab Corp., USA).

## Results

### Effects of trap translucency

**Fig 2:**
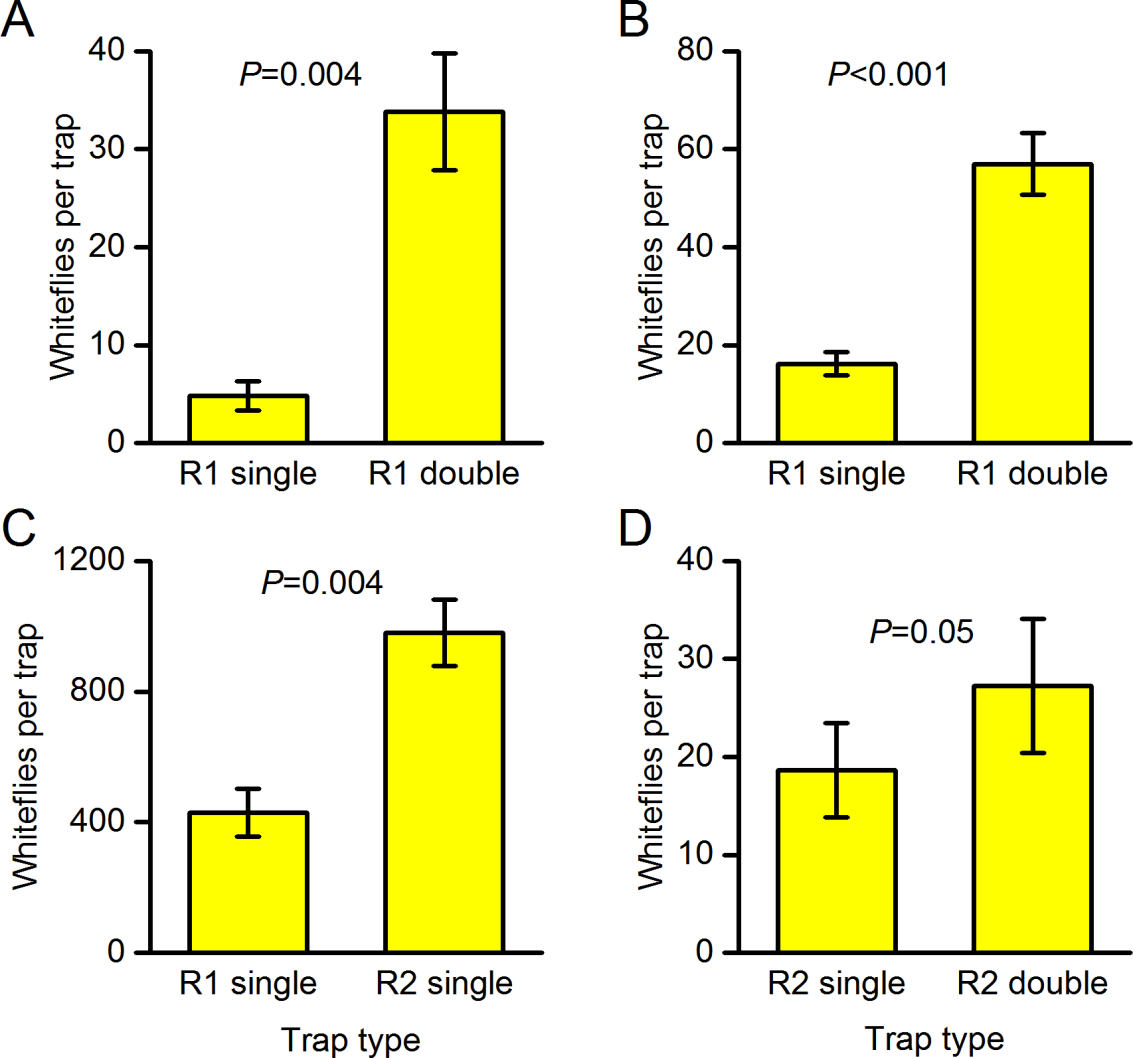
Effects of trap translucency on the number of whiteflies caught on sections of yellow sticky roller trap. Mean catch on both sides of the trap ± SE. (A) Experiment A in 2016 comparing single and double thicknesses of roller trap type R1 (moderate translucency) (*n* = 6), (B) experiment A repeated in 2017 (*n* = 9), (C) experiment B comparing single thicknesses of roller trap type R1 (moderate translucency) with roller trap type R2 (less translucency) (*n* = 9), (D) experiment C comparing single and double thicknesses of roller trap type R2 (less translucency) (*n* = 8). Differences in trap catch between experiments reflect trap area, crop infestation and the duration of each experiment, so comparisons of treatments between experiments are not valid.

Experiment A (thickness of trap type R1). Doubling the thickness of a yellow sticky roller trap with moderate translucency (trap type R1) significantly increased the whitefly trap catch (*F*_(1,5)_ = 24.3, *P* = 0.004) by a factor of 7.0 (Fig 2A). The experiment was repeated a year later and doubling the thickness again increased the whitefly trap catch (*F*_(1,8)_ = 52.7, *P*<0.001), by a factor of 3.5 (Fig 2B). The catch of females was increased (*F*_(1,8)_ = 22.0, *P* = 0.002) by a factor of 3.9 and the catch of males was increased (*F*_(1,8)_ = 57.5, *P*<0.001) by a factor of 3.4. Whiteflies were caught mainly near the bottom edge of the roller traps, nearest the crop, both on single and double thickness traps.

Experiment B (trap types R1 and R2). Traps that were less translucent (trap type R2) significantly increased the whitefly trap catch compared with traps with moderate translucency (trap type R1) (*F*_(1,8)_ = 15.7, *P* = 0.004) by a factor of 2.3 (Fig 2C).

Experiment C (thickness of trap type R2). Doubling the thickness of the less translucent traps (trap type R2) caught more whiteflies, by a factor of 1.5 (Fig 2D), but the difference was marginally not significant (*F*_(1,7)_ = 5.5, *P* = 0.052).

**Fig 3:**
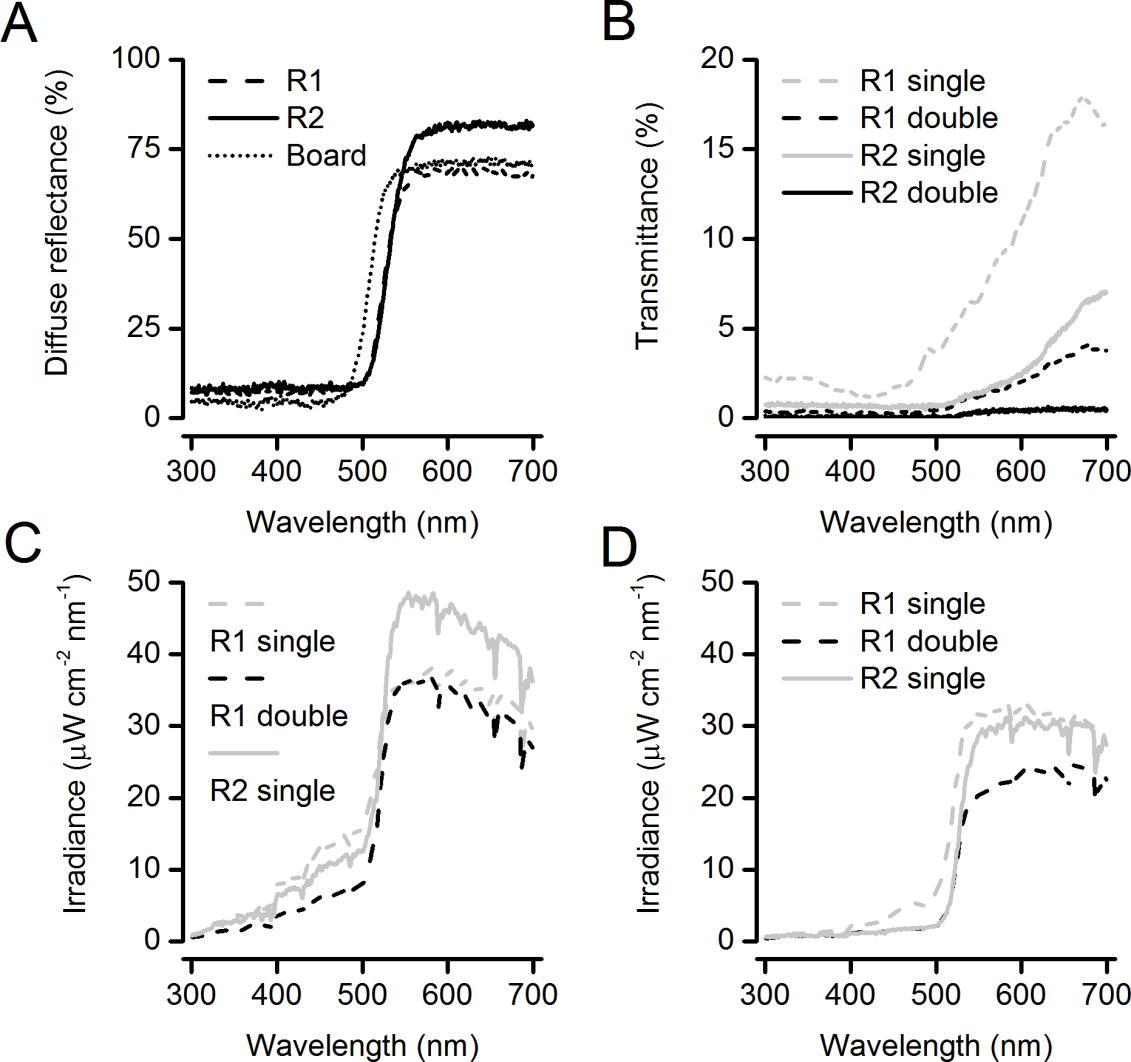
Diffuse reflectance, transmittance (translucency) and irradiance of traps. (A) Percentage diffuse reflectance of roller trap type R1 (R1), roller trap type R2 (R2) and the yellow board trap (Board); (B) percentage transmittance of roller traps; (C) absolute irradiance of roller traps with the sun shining on the front (sensor side); (D) absolute irradiance of traps with the sun shining on the back (other side from sensor). Key: R1 single = roller trap type R1 single thickness; R1 double = roller trap type R1 double thickness; R2 single = roller trap type R2 single thickness; and R2 double = roller trap type R2 double thickness.

Diffuse reflectance measurements (Fig 3A) showed that the yellows of the two roller traps (types R1 and R2) and the board traps were similar, with low diffuse reflectance below 500 nm and a high diffuse reflectance above 500 nm. The slightly higher diffuse reflectance above 500 nm of the less translucent roller trap (type R2) is probably a result of the higher pigment density. Transmittance spectra (Fig 3B) showed, as expected from observation, that double thickness traps were less translucent (transmitted less light) than single thickness traps and that trap type R2 was less translucent than trap type R1. The transmittance increased steadily over the range from 500–700 nm and so was dominated by longer wavelengths well above 500 nm.

Absolute irradiance measurements of the traps when the sun was shining on the front (the sensor side) were dominated by the diffuse reflectance from the front. Doubling the thickness of trap type R1 decreased the wavelengths below 500 nm relative to the wavelengths above 500 nm (Fig 3C) and the same effect was seen when comparing trap type R1 with the less translucent trap type R2. Measurements when the sun was shining on the back of the traps (the other side from the sensor) had both diffuse reflectance and transmitted light (translucency) from behind. The same effect was observed as when the sun was shining on the front of the traps (Fig 3D), with the thicker or less translucent traps decreasing the wavelengths below 500 nm relative to the wavelengths above 500 nm (Fig 3D).

We can test whether the colour opponency mechanism predicts the relative attraction of the yellow traps by calculating the ratio of light energy in the range 500–640 nm to that in the range 300–500 nm (see Introduction). The values can be obtained from the areas under the curve of the absolute irradiance spectra (Fig 3C and 3D). It is only an approximate measure because we do not have detailed spectral sensitivities of the whiteflies or records of the irradiances when the whiteflies were caught. Higher energy ratios (*ER*) would be expected to catch more whiteflies. The energy ratios were higher for the trap types that caught more in the three experiments where there was a significant difference in trap catch (Fig 2A–2C): trap type R1 single thickness (*ER* = 3.1 sun on front, 7.9 sun on back), trap type R1 double thickness (*ER* = 6.0 sun on front, 11.7 sun on back) and trap type R2 single thickness (*ER* = 4.7 sun on front, 14.8 sun on back). Inspection of the spectral irradiance curves (Fig 3C and 3D) indicated that analysis of narrower energy ranges closer to the known whitefly sensitivity peaks above 500 nm (520 nm) and below 500 nm (340 nm) would give similar results. The trap catches in experiments A-C cannot be compared between the side with the sun on it and the side with the sun shining through because the position of the sun changed throughout the experiments and was not shining consistently on one side.

### Effects of trap patterns

Pilot studies suggested that yellow board traps with a high proportion of the area covered with black pattern often decreased trap catches or had no effect, so our experiments generally used traps with a black pattern on no more than about 30% of the area of one side. In the experiments below, the ‘front’ refers to the pattern side on pattern traps and the side facing the same direction on plain traps, whereas the ‘back’ of the trap refers to the non-printed side of pattern traps and the side facing the same direction on plain traps.

**Fig 4:**
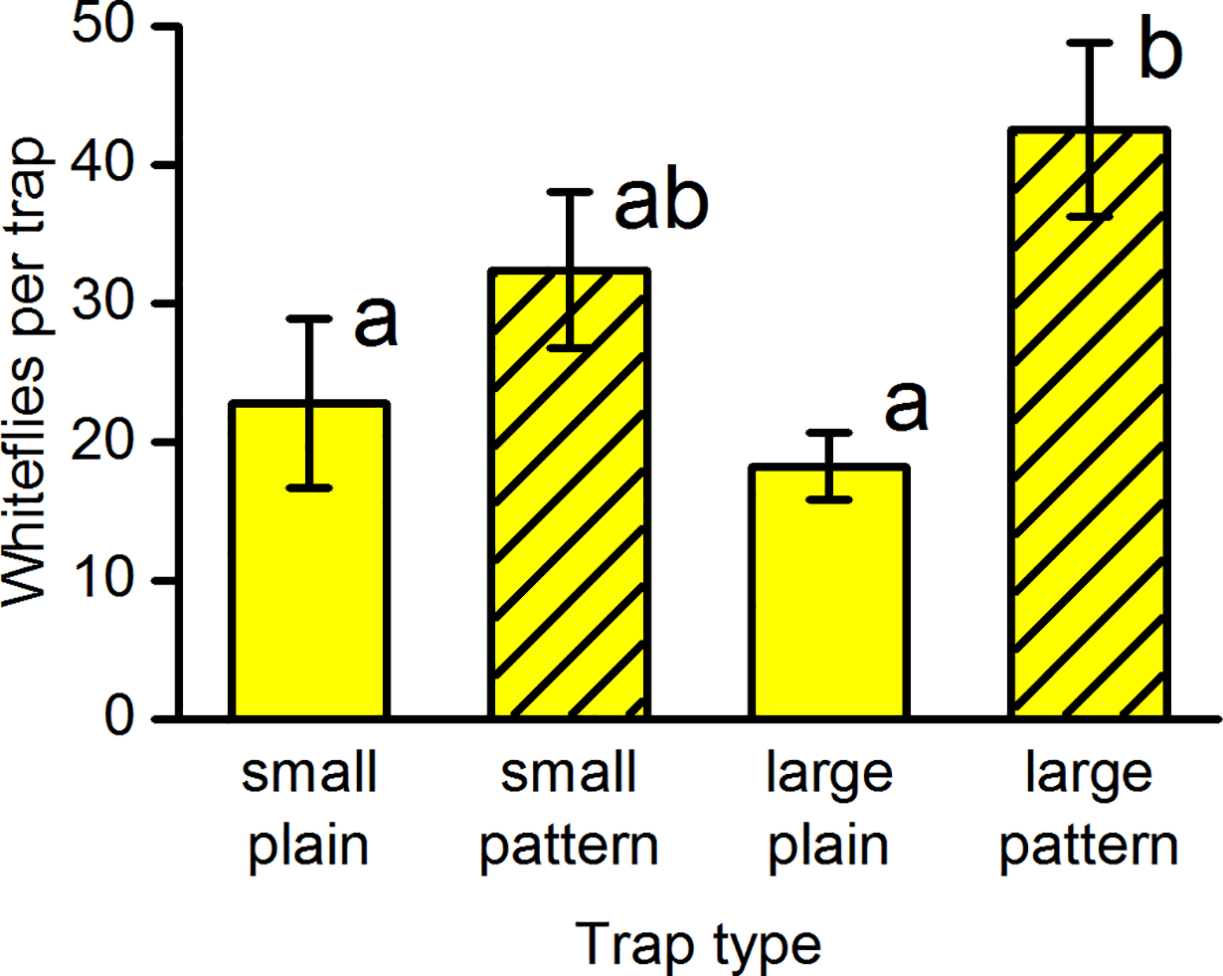
Effects of trap size and pattern on the number of whiteflies caught on yellow sticky board traps. Mean catch on both sides of the trap ± SE in experiment D (*n* = 20). Key: small = 10 cm wide x 12.5 cm high; large = 10 cm wide x 25 cm high; plain = no pattern; pattern = pattern of uniform ovals (pattern A). Bars with the same letter are not significantly different (*P*>0.05) from each other using Tukey’s test on log-transformed data allowing for blocks.

Experiment D (pattern and trap size). The effect of a printed black pattern was tested and compared between small (10 cm x 12.5 cm) and large (10 cm x 25 cm) yellow sticky traps. There was a significant difference in catch between the four combinations of two trap sizes with and without pattern (*F*_(3,57)_ = 5.4, *P* = 0.002) (Fig 4). The pattern significantly increased trap catch on the large traps by a factor of 2.3, but was not significant on the small traps. Analysis by orthogonal contrasts showed a highly significant overall effect of pattern (*F*_(1,57)_ = 14.5, *P*<0.001), which was significant on the front of the trap (*F*_(1,57)_ = 12.2, *P* = 0.001) as well as on the back (*F*_(1,57)_ = 7.3, *P* = 0.009). There was no significant overall effect of trap size (*F*_(1,57)_ = 1.2, *P* = 0.28) and the interaction between trap size and pattern was not significant (*F*_(1,57)_ = 0.64, *P* = 0.43). The large traps were the same width as the small traps but reached further up away from the crop. When the catch of whiteflies is mainly on the part of the trap nearer the crop, the area of trap higher above the crop makes little difference.

Experiment E (pattern and distribution of catch). The pattern significantly increased the overall whitefly trap catch (*F*_(1,16)_ = 20.5, *P*<0.001) by a factor of 1.4 (Table 1). The increase was significant on the front of the traps (*F*_(1,16)_ = 7.2, *P* = 0.02), as well as on the back of the traps (*F*_(1,16)_ = 22.9, *P*<0.001). The trap catch ratio between pattern traps and plain traps was greatest in the immediate vicinity of the pattern (front middle) and where the pattern showed through, appearing greyish-yellow (back middle) (Table 1). Whiteflies on the traps were sometimes observed to be aligned along the edges of the black pattern, either on the printed front or where the pattern showed through to the back.

**Table 1:** The distribution on traps of whiteflies caught on yellow sticky traps with and without patterns in experiment E.

Trap side	Position on trap	Mean whiteflies (±SE) on pattern traps	Mean whiteflies (±SE) on plain traps	Whitefly ratio (pattern:plain)
**Front**	**Top**	18.0 ± 3.2	17.2 ± 3.4	1.0
	**Middle**	26.4 ± 3.1	12.1 ± 2.9	2.2
	**Bottom**	14.4 ± 2.2	17.7 ± 3.3	0.8
	**Total**	58.8 ± 8.0	47.1 ± 9.3	1.2 (*P* = 0.02)
**Back**	**Top**	30.4 ± 5.6	25.6 ± 4.8	1.2
	**Middle**	44.4 ± 7.0	17.9 ± 3.6	2.5
	**Bottom**	24.9 ± 4.2	23.6 ± 4.6	1.1
	**Total**	99.7 ± 16.5	67.1 ± 12.4	1.5 (*P*<0.001)
**Both sides**		158.5 ± 23.9	114.2 ± 20.7	1.4 (*P*<0.001)

Pattern traps had a pattern printed on the front middle of the trap. The pattern showed through, appearing greyish-yellow, on the back middle of the pattern traps. The top, middle and bottom parts had equal areas of glue. The *P*-values are calculated by ANOVA with log-transformed data comparing pattern traps with plain traps (*n* = 17).

**Fig 5:**
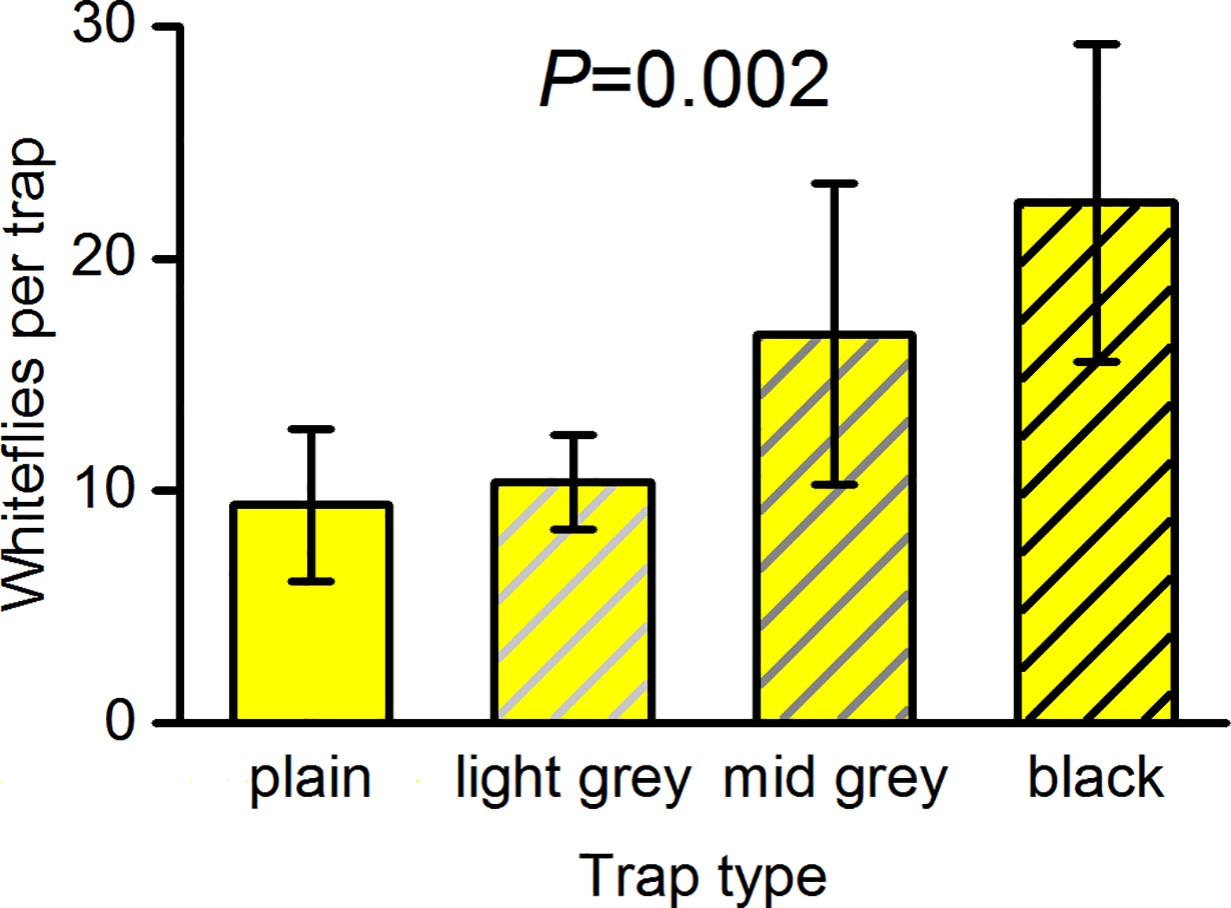
Effects of patterns with increasing shades of grey on the number of whiteflies caught on yellow sticky board traps. Mean catch on the pattern side and corresponding side of plain (control) traps ± SE in experiment F (*n* = 20). The significance is from a regression of log-transformed trap catch on darkness of grey/black.

Experiment F (pattern and shade of grey). Trap catch increased with increasing shades of grey from no pattern through light grey and mid grey to black (general linear model regression, *F*_(1,59)_ = 10.4, *P* = 0.002) (Fig 5). Comparison of the plain yellow traps with the yellow traps with the black pattern for each sex separately showed that there was a significant increase on the pattern traps for females (*F*_(1,19)_ = 6.4, *P* = 0.02) by a factor of 2.5 and for males (*F*_(1,19)_ = 4.5, *P* = 0.048) by a factor of 2.3.

The black pattern occupied a small proportion (5%) of the trap area and had a negligible effect on the overall absolute irradiance. The black pattern itself gave minimal diffuse reflectance. Where the pattern showed through to the back as greyish yellow, its diffuse reflectance spectrum was a less reflective version of the spectrum for yellow across all wavelengths.

### Effects of trap translucency on response to trap pattern

**Fig 6:**
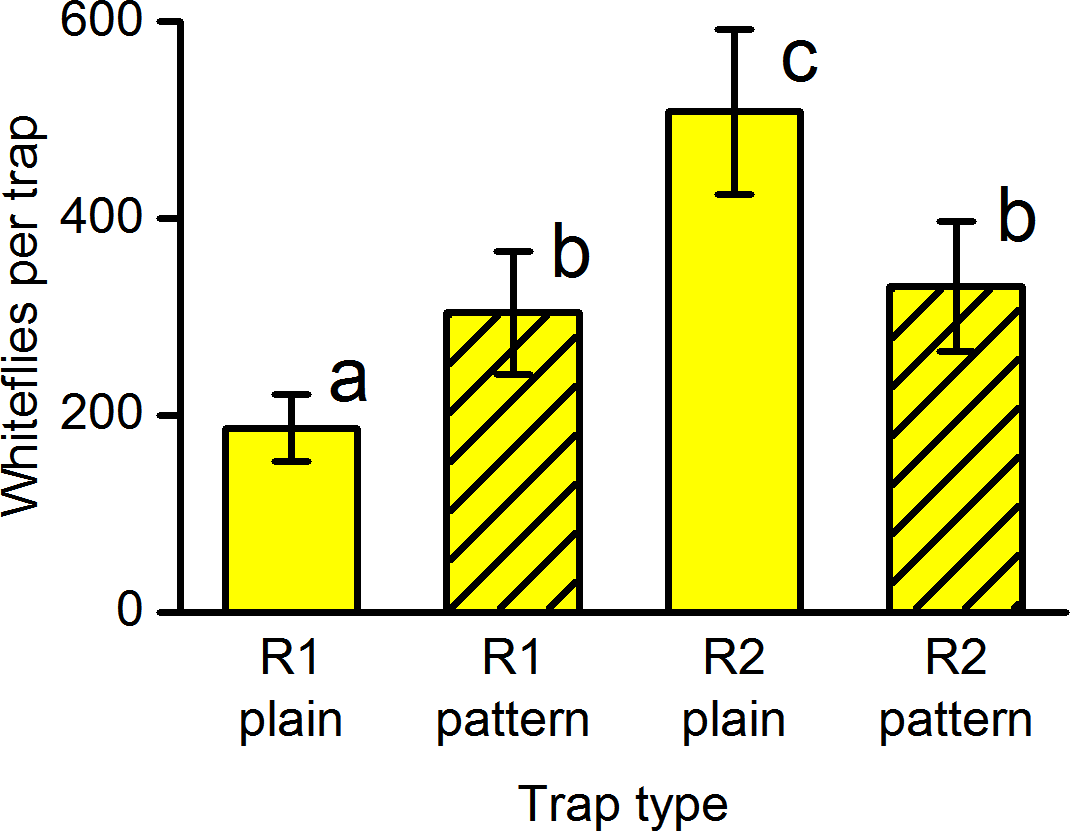
Effects of trap translucency and pattern on the number of whiteflies caught on sections of yellow sticky roller trap. Mean catch on both sides of the trap ± SE in experiment G (*n* = 10). R1 = roller trap type R1 (moderate translucency), R2 = roller trap type R2 (less translucency). Bars with the same letter are not significantly different (*P*>0.05) from each other using Tukey’s test on log-transformed data allowing for blocks.

Experiment G (translucency and pattern). There was a highly significant difference between the combinations of two roller trap types with and without pattern (*F*_(3,27)_ = 19.5, *P*<0.001) (Fig 6). The pattern increased trap catch on the moderately translucent trap (type R1), but decreased trap catch on the less translucent trap (type R2). Analysis by orthogonal contrasts showed a significant interaction between trap type and presence/absence of pattern (*F*_(1,27)_ = 20.1, *P*<0.001). There was a significant overall effect of trap type (*F*_(1,27)_ = 38.3, *P*<0.001) and no significant overall effect of pattern (*F*_(1,27)_ = 0.0, *P* = 0.96), but these main effects should be interpreted with caution because of the significant cross-over interaction between trap type and pattern (Fig 6).

## Discussion

Although all the yellow roller traps appeared a very similar yellow to the human eye, small changes in translucency had large effects on the number of whiteflies caught on traps in commercial crops. Reduced translucency increased total catch by a factor ranging from 1.5 to 7.0 and was significant for both females and males. These effects could improve the efficacy of traps for monitoring and mass trapping of *T*. *vaporariorum* in crops.

Spectrometer measurements of energy ratios were consistent with an increased landing stimulus from a colour opponency mechanism (see Introduction) to explain the differences in trap catch between similar yellows. An alternative explanation could be that whiteflies are more attracted to less bright traps, since the double thickness traps generally had a lower overall absolute irradiance than the single thickness traps (Fig 3C and 3D). However, this seems unlikely as the difference in overall brightness is small compared with the enormous variation in the brightness of daylight throughout the day, and yellow traps are not more effective in the shade than in the sun.

How does yellow trap translucency alter the relative amounts of energy above and below 500 nm? Diffuse reflectance is not just from the surface of a material but also from subsurface scattering in translucent materials [40], so a thicker or more densely pigmented trap will scatter more light and act as a more effective filter for diffuse reflectance. In this case, as seen in the diffuse reflectance spectra (Fig 3A), wavelengths below 500 nm were filtered out more than wavelengths above 500 nm for thicker or more densely pigmented yellow traps. The transmittance spectra (Fig 3B) also showed that thicker or more densely pigmented yellow traps filtered out relatively more of the wavelengths below 500 nm for light from behind the trap. Thus, both the diffuse reflectance from the front and transmitted light from behind contributed to the effect of higher energy ratios in thicker or more densely pigmented traps. These translucency effects could also affect the trap catch of other whiteflies, such as *B*. *tabaci*, or other insects monitored with yellow traps, such as aphids, thrips and leafminers, and it would be interesting to test this in the future.

Despite the widespread use of yellow traps, the ways in which the appearance of a yellow trap changes through the day and is affected by factors such as the position of the sun and specular reflection have received little attention. Better understanding of yellow traps and how insects respond to them could improve them further by increasing the catch of pest species and reducing the catch of beneficial insects.

Printed black patterns also increased trap catches in most cases, by a factor of about 1.4 to 2.3, and the catch increased for both females and males. The evidence points to a visual effect not an odour effect. Plant odours are known to play a role in the orientation and landing of *T*. *vaporariorum*, and visual and odour cues could have a synergistic effect [41,42]. However, the possibility that the odour of the black ink was responsible for the attraction can be rejected because in experiment F the catch was affected by the shade of ink even though the same amount of ink was used on each trap and in experiment G the presence of the ink significantly decreased trap catch for one treatment.

Experiment E showed that the patterns increased the catch in the immediate vicinity of the pattern or on the back of the trap where the pattern showed through. The amount of show-through is determined by the position of the sun, and as this changed throughout the experiment, the result is difficult to interpret. Since the pattern side faced east in this experiment, the show-through would have been greatest in the morning when the sun was in the east. The little information available about diel flight patterns of *T*. *vaporariorum* in greenhouses suggests that flight activity may be greater in the morning [43].

The reason that black patterns increased trap catch remains unclear. Our experiments do not distinguish between whether it is due to the stimulating effect of contrasting edges or an optomotor response to the black lines (see Introduction). Several observations point to a contrast/edge effect: (1) black patterns had more effect than grey patterns; (2) the catch was in the immediate vicinity of the patterns; and (3) whiteflies were sometimes caught particularly along the edges of the black patterns, either on the front of the trap or on the back where the pattern showed through and in the narrow margins between the pattern and the sides of board traps. Now that this effect has been shown to be relevant in the field, laboratory experiments are needed to observe the landing responses under controlled conditions.

Black patterns are known to attract house flies *Musca domestica* (L.) to sticky traps [44,45], but the explanation that the patterns resemble groups of flies or cracks for harbourage are unlikely to apply to whiteflies.

Experiment G showed that patterns increased the catch on a moderately translucent trap (R1), but decreased the catch on a less translucent trap (R2). This unexpected result has been confirmed by further experiments. Why should a change in translucency reverse the effect of the pattern? We speculate that there are two opposing effects. The first effect is increased attraction by increasing the contrast between the black and the yellow. Thus a more translucent trap would increase the brightness of the yellow area within the pattern and so increase the contrast with the black pattern. Increased contrast is probably also the reason why black patterns caught more than grey patterns (experiment F). The second effect is a reduction in trap catch as a result of replacement of attractive yellow by non-attractive black [30]. A change in translucence could change the balance between these positive and negative effects. Again, laboratory experiments should be able to elucidate these effects.

## Supporting information

S1 File Spreadsheet with raw data from experiments A-F.
(XLSX)

## References

[1] Pinto-Zevallos DM, Vänninen I (2013) Yellow sticky traps for decision-making in whitefly management: What has been achieved? Crop Prot 47:74–84.

[2] Lu Y, Bei Y, Zhang J (2012) Are yellow sticky traps an effective method for control of sweetpotato whitefly, *Bemisia tabaci*, in the greenhouse or field? J Insect Sci 12(113):1–12. 10.1673/031.012.11301 PMC3620036 23445077

[3] Böckmann E, Hommes M, Meyhoefer R (2015) Yellow traps reloaded: what is the benefit for decision making in practice? J Pest Sci 88:439–449.

[4] Shimoda M, Honda K (2013) Insect reactions to light and its applications to pest management. Appl Entomol Zool 48:413–421.

[5] Disney RHL, Erzinçlioglu YZ, Henshaw DJD, Howse D, Unwin DM, Withers P, et al (1982) Collecting methods and the adequacy of attempted fauna surveys with reference to the Diptera. Field Stud 5:607–622.

[6] Vrdoljak SM, Samways MJ (2012) Optimising coloured pan traps to survey flower visiting insects. J Insect Conserv 16:345–354.

[7] Lloyd L (1921) Notes on a colour tropism of *Asterochiton* (*Aleurodes*) *vaporariorum*, *Westwood*. Bull Entomol Res 12:355–359.

[8] Prokopy RJ, Owens ED (1983) Visual detection of plants by herbivorous insects. Annu Rev Entomol 28:337–364.

[9] Kirk WDJ (1984) Ecologically selective coloured traps. Ecol Entomol 9:35–42.

[10] Bernays EA, Chapman RF (1994) Behavior: the process of host-plant selection. Chapter 4. In: Host-Plant Selection by Phytophagous Insects London: Chapman & Hall pp. 95–165.

[11] Döring TF, Chittka L (2007) Visual ecology of aphids—a critical review on the role of colours in host finding. Arthropod-Plant Interact 1:3–16.

[12] Hardie J (1989) Spectral specificity for targeted flight in the black bean aphid *Aphis fabae*. J Insect Physiol 35:619–626.

[13] Kennedy JS, Booth CO, Kershaw WJS (1961) Host finding by aphids in the field. III. Visual attraction. Ann Appl Biol 49:1–21.

[14] Stukenberg N, Gebauer K, Poehling H-M (2015) Light emitting diode (LED)-based trapping of the greenhouse whitefly (*Trialeurodes vaporariorum*). J Appl Entomol 139:268–279.

[15] Vernon RS, Gillespie DR (1990) Spectral responsiveness of *Frankliniella occidentalis* (Thysanoptera: Thripidae) determined by trap catches in greenhouses. Environ Entomol 19:1229–1241.

[16] Vernon RS, Gillespie DR (1990) Response of *Frankliniella occidentalis* (Thysanoptera: Thripidae) and *Trialeurodes vaporariorum* (Homoptera: Aleyrodidae) to fluorescent traps in a cucumber greenhouse. J Entomol Soc B C 87:38–41.

[17] Döring TF, Röhrig K (2016) Behavioural response of winged aphids to visual contrasts in the field. Ann Appl Biol 168:421–434.

[18] Vernon RS, Gillespie DR (1995) Influence of trap shape, size, and background color on captures of *Frankliniella occidentalis* (Thysanoptera: Thripidae) in a cucumber greenhouse. J Econ Entomol 88:288–293.

[19] Kim S, Lim UT (2011) Evaluation of a modified sticky card to attract *Bemisia tabaci* (Hemiptera: Aleyrodidae) and a behavioural study on their visual response. Crop Prot 30:508–511.

[20] Mainali BP, Lim UT (2010) Circular yellow sticky trap with black background enhances attraction of *Frankliniella occidentalis* (Pergande) (Thysanoptera: Thripidae). Appl Entomol Zool 45:207–213.

[21] Costa CL, Lewis T (1967) The relationship between the size of yellow water traps and catches of aphids. Entomol Exp Appl 10:485–487.

[22] Kirk WDJ (1987) Effects of trap size and scent on catches of *Thrips imaginis* Bagnall (Thysanoptera: Thripidae). J Aust Entomol Soc 26:299–302.

[23] Kaas JP (2005) Vertical distribution of thrips and whitefly in greenhouses and relative efficiency of commercially available sticky traps for population monitoring. Proc Exp Appl Entomol 16:109–116.

[24] Gillespie DR, Vernon RS (1990) Trap catch of western flower thrips (Thysanoptera: Thripidae) as affected by color and height of sticky traps in mature greenhouse cucumber crops. J Econ Entomol 83:971–975.

[25] Gillespie DR, Quiring DJM (1992) Flight behavior of the greenhouse-whitefly, *Trialeurodes vaporariorum* (Westwood) (Homoptera, Aleyrodidae), in relation to yellow sticky traps. Can Entomol 124:907–916.

[26] Affeldt HA, Thimijan RW, Smith FF, Webb RE (1983) Response of the greenhouse-whitefly (Homoptera, Aleyrodidae) and the vegetable leafminer (Diptera, Agromyzidae) to photospectra. J Econ Entomol 76:1405–1409.

[27] Chu CC, Jackson CG, Alexander PJ, Karut K, Henneberry TJ (2003) Plastic cup traps equipped with light-emitting diodes for monitoring adult *Bemisia tabaci* (Homoptera: Aleyrodidae). J Econ Entomol 96:543–546. 12852586 10.1603/0022-0493-96.3.543

[28] Byrne DN, Bellows TS, Parrella MP (1990) Whiteflies in agricultural systems In: Gerling D, editor. Whiteflies: Their Bionomics, Pest Status and Management. Andover: Intercept pp 227–261.

[29] Gratwick M (1992) Crop Pests in the UK: Collected Edition of MAFF Leaflets. London: Chapman & Hall pp. 490.

[30] Vaishampayan SM, Kogan M, Waldbauer GP, Woolley JT (1975) Spectral specific responses in the visual behavior of the greenhouse whitefly *Trialeurodes vaporariorum* (Homoptera: Aleyrodidae). Entomol Exp Appl 18:344–356.

[31] Webb RE, Smith FF, Affeldt H, Thimijan RW, Dudley RF, Webb HF (1985) Trapping greenhouse whitefly with coloured surfaces: variables affecting efficacy. Crop Prot 4:381–393.

[32] Coombe PE (1981) Wavelength specific behavior of the whitefly *Trialeurodes vaporariorum* (Homoptera: Aleyrodidae). J Comp Physiol A 144:83–90.

[33] MacDowall FD (1972) Phototactic action spectrum for whitefly and question of color-vision. Can Entomol 104:299–307.

[34] Mellor HE, Bellingham J, Anderson M (1997) Spectral efficiency of the glasshouse whitefly *Trialeurodes vaporariorum* and *Encarsia formosa* its hymenopteran parasitoid. Entomol Exp Appl 83:11–20.

[35] Coombe PE (1981) Visual behaviour of the whitefly Trialeurodes vaporariorum (Westwood) (Homoptera: Aleyrodidae). University of Adelaide: PhD thesis.

[36] Moericke V, Schneiders H, Vogt B (1966) Flughemmung und Fallreflexhaltung als Reaktion auf Gelbreiz bei *Trialeurodes vaporariorum* (Westwood). Z Pflanzenkrankh Pflanzenpathol Pflanzenschutz 73:6–14.

[37] Horridge A (2009) What does an insect see? J Exp Biol 212:2721–2729. doi: 10.1242/jeb.030916 19684204 10.1242/jeb.030916

[38] Steiner MY, Spohr LJ, Barchia I, Goodwin S (1999) Rapid estimation of numbers of whiteflies (Hemiptera: Aleurodidae) and thrips (Thysanoptera: Thripidae) on sticky traps. Aust J Entomol 38:367–372.

[39] Walker GP, Perring TM, Freeman TP (2010) Life history, functional anatomy, feeding and mating behavior. Chapter 4 In: Stansly PA, Naranjo SE, editors. *Bemisia*: Bionomics and Management of a Global Pest. Dordrecht: Springer pp 109–160.

[40] Hanrahan P, Krueger W (1993) Reflection from layered surfaces due to subsurface scattering. Proc 20th Annu Conf Comput Graph Interact Tech 1993:165–174.

[41] Darshanee HLC, Ren H, Ahmed N, Zhang ZF, Liu YH, Liu TX (2017) Volatile-mediated attraction of greenhouse whitefly *Trialeurodes vaporariorum* to tomato and eggplant. Front Plant Sci 8:e1285. 10.3389/fpls.2017.01285 PMC5517405 28775733

[42] Vaishampayan SM, Waldbauer GP, Kogan M (1975) Visual and olfactory responses in orientation to plants by the greenhouse whitefly *Trialeurodes vaporariorum* (Homoptera:Aeyrodidae). Entomol Exp Appl 18:412–422.

[43] Liu T-X, Oetting RD, Buntin GD (1994) Temperature and diel catches of *Trialeurodes vaporariorum* and *Bemisia tabaci* (Homoptera: Aleyrodidae) adults on sticky traps in the greenhouse. J Entomol Sci 29:222–230.

[44] Chapman JW, Knapp JJ, Goulson D (1999) Visual responses of *Musca domestica* to pheromone impregnated targets in poultry units. Med Vet Entomol 13:132–138. 10484159 10.1046/j.1365-2915.1999.00147.x

[45] Diclaro JW, Cohnstaedt LW, Pereira RM, Allan SA, Koehler PG (2012) Behavioral and physiological response of *Musca domestica* to colored visual targets. J Med Entomol 49:94–100. 22308776 10.1603/me10257

